# Natural History of Skeletal Muscle Mass Changes in Chronic Kidney Disease Stage 4 and 5 Patients: An Observational Study

**DOI:** 10.1371/journal.pone.0065372

**Published:** 2013-05-31

**Authors:** Stephen G. John, Mhairi K. Sigrist, Maarten W. Taal, Christopher W. McIntyre

**Affiliations:** 1 Department of Renal Medicine, Royal Derby Hospital, Derby, United Kingdom; 2 School of Graduate Entry Medicine and Health, University of Nottingham, Derby, United Kingdom; Mario Negri Institute for Pharmacological Research and Azienda Ospedaliera Ospedali Riuniti di Bergamo, Italy

## Abstract

Cross-sectional studies in dialysis demonstrate muscle wasting associated with loss of function, increased morbidity and mortality. The relative drivers are poorly understood. There is a paucity of data regarding interval change in muscle in pre-dialysis and dialysis-dependant patients. This study aimed to examine muscle and fat mass change and elucidate associations with muscle wasting in advanced CKD.

134 patients were studied (60 HD, 28 PD, 46 CKD 4–5) and followed up for two years. Groups were similar in age, sex and diabetes prevalence. Soft tissue cross-sectional area (CSA) was measured annually on 3 occasions by a standardised multi-slice CT thigh. Potential determinants of muscle and fat CSA were assessed. Functional ability was assessed by sit-to-stand testing.

88 patients completed follow-up (40 HD, 16 PD, 32 CKD). There was a significant difference in percentage change in muscle CSA (MCSA) over year 1, dependant on treatment modality (χ^2^ = 6.46; p = 0.039). Muscle loss was most pronounced in pre-dialysis patients. Muscle loss during year 1 was partially reversed in year 2 in 39%. Incident dialysis patients significantly lost MCSA during the year which they commenced dialysis, but not the subsequent year. Baseline MCSA, change in MCSA during year 1 and dialysis modality predicted year 2 change in MCSA (adjusted R^2^ = 0.77, p<0.001). There was no correlation between muscle or fat CSA change and any other factors. MCSA correlated with functional testing, although MCSA change correlated poorly with change in functional ability.

These data demonstrate marked variability in MCSA over 2 years. Loss of MCSA in both pre-dialysis and established dialysis patients is reversible. Factors previously cross-sectionally shown to correlate with MCSA did not correlate with wasting progression. The higher rate of muscle loss in undialysed CKD patients, and its reversal after dialysis commencement, suggests that conventional indicators may not result in optimal timing of dialysis initiation.

## Introduction

Significant muscle atrophy [1], [2] and associated weakness [3] is seen in both dialysis patients and in patients with chronic kidney disease (CKD) stages 3–4. This is associated with increased morbidity and mortality [4], [5].

There are many associations already reported as being associated on a cross sectional study basis with muscle wasting. These include decreasing glomerular filtration rate (GFR) [6], dialysis [7], age [8] and diabetes [9], [10]. Other key drivers of muscle mass in CKD include exercise, acidosis and insulin resistance [11], [12] as well as inflammation [12]. Muscle atrophy can be assessed via various methods, and previous work in our group has shown good correlation between muscle cross-sectional area and functional performance, serum albumin, age and inflammatory status in CKD stages 4–5, haemodialysis (HD) and peritoneal dialysis (PD) [13]. More recently, reduced muscle and fat mass evidenced by anthropomorphic assessment (mid-arm circumference, triceps skinfold) appears able to predict mortality in haemodialysis populations [14], [15].

These changes have previously been attributed to inadequate dietary protein intake, but recent advances in our understanding of this area indicate other processes are involved. Animal models have suggested that the ubiquitin-proteasome system, the protease caspase-3 [16] and suppression of phosphatidylinositol 3-kinase (PI3-K) activity [17] may be responsible for accelerated muscle breakdown in CKD. Recent work has increased the focus on these key pathways in humans [11], [12], [18]. The triggers are unclear, although IGF-1 is implicated. Insulin resistance is common and is associated with muscle wasting in non-diabetic dialysis patients [19], [20], [21]. Muscle turnover increases during HD, resulting in net increase in catabolism [22]. Finally, electromyography (EMG) studies have suggested that circulating uraemic toxins depress muscle function, and this improves acutely with haemodialysis [23].

Muscle mass can be modulated by exercise training [7], [24], [25], [26], [27], which improves both muscle structure and function in dialysis patients. Also, anabolic steroids increase thigh muscle cross-sectional area in an additive manner to resistance exercise training in this group [28]. Finally, L-carnitine supplementation may be effective in some dialysis patients [29].

There is significantly less literature concerning the role of fat. Fat mass in haemodialysis patients correlates with increasing age [8] and diabetic haemodialysis patients have more thigh adipose tissue than non-diabetics [7]. In haemodialysis patients, low baseline body fat percentage and fat loss over time are independently associated with higher mortality [30]. Much of the available data regarding body fat in CKD has been generated as part of work on the malnutrition-inflammation-atherosclerosis (MIA) complex, as the contribution of adipose tissue to inflammation via adipokine (including leptin and adiponectin) and cytokine (including TNFα and IL-6) secretion [31] is becoming further understood.

## Methods

### Objectives

The aim of this study was to examine the interval muscle and fat change, predictors of such change, and associated functional abnormalities in a cohort of pre-dialysis CKD, HD and PD patients.

### Participants

We studied 134 subjects (46 CKD stage 4, 60 HD, 28 PD) recruited from Derby City General Hospital [13]. Exclusion criteria were previous transplantation, limb amputation or conditions likely to increase muscle catabolism including active sepsis or malignancy. All prevalent patients were approached (291 patients in May 2003, at the start of recruitment). CKD stage 4 patients were defined by a minimum of two eGFR (MDRD) measurements of 15–30 ml/min. Dialysis modality was subject to patient choice, no patient had switched modality during the study. All dialysis patients were established for at least 6 months.

PD patients were all treated with bicarbonate/lactate buffered fluid (Physioneal®, Baxter, UK). 9 patients used automated PD, 19 patients used continuous ambulatory PD (3–5 exchanges/day).

HD patients received three sessions of at least 4 hours per week. Hospal Integra (Mirandola, Italy) monitors and low-flux polysulphone dialysers (1.5–2.0 m^2^, LOPS 15–20®, Braun Medical Ltd, Sheffield, UK). Dialysate containing 1.25 mEq/l calcium and 134 mmol/l sodium was used in all sessions. All sessions utilised bicarbonate-based HD.

Standard dietary advice and support was provided to all subjects as standard policy. Daily sodium intake was limited to 100 mmol, and daily protein to 1.2 g/kg for at least 1 year prior to study.

### Description of Procedures and Investigations

**Figure 1:**
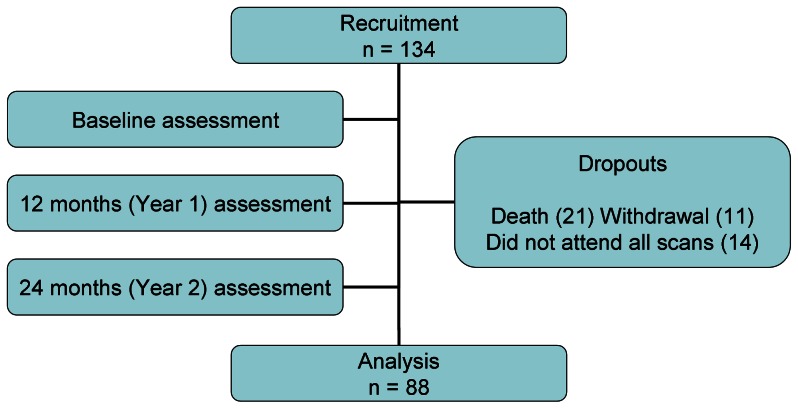
Study diagram.

Participants were studied at baseline, 12 months and 24 months (Figure 1).

### Data collection

Age, gender, comorbiditity, medication, height, weight and tobacco and alcohol use were recorded for all patients. Biochemical parameters (haemoglobin, serum phosphate, serum corrected calcium, albumin (bromocresol purple method) and serum bicarbonate) were time-averaged over the previous 6 months. High sensitivity CRP was assayed at each investigatory visit by ELISA (DRG Diagnostics, Marburg, Germany). The most recent (less than one month before each visit) dialysis adequacy was recorded.

### Imaging

Muscle and fat CSA was assessed by computed tomography utilising a GE Medical Systems LightSpeed16 multi-slice spiral scanner. Non-contrast enhanced images were obtained from supine subjects. A standardised 6 cm section of thigh was scanned, 20 cm above the tibial plateau, in 2.5 mm slices without overlap. Total radiation exposure was calculated to be 116 mgy, lower than a standard chest X-ray (120 mgy).

Scans were independently scored by two investigators, blinded to patient group and identity, using GE Medical Systems Advantage Workstation software (intra-observer CoV of <1%).. Muscle and fat CSA was measured by drawing an appropriate region of interest around the images with windowing for the appropriate density (fat and non-contractile connective tissue -200-0 Hounsfield units (HU), muscle 0-200 HU). The mean of the top, middle and bottom images was taken, and then adjusted for patient height [13]. This technique aimed to create a comparable and repeatable assessment between patients allowing for differing patient morphology.

### Functional muscle assessment

Physical function was assessed using sit-to-stand (STS) testing. Subjects were timed rising from sitting in a chair of standard height (46 cm) and returning to sitting. This was demonstrated prior to assessment. Both muscle power (STS 5 – time to perform 5 iterations) and endurance (STS 60 – maximum iterations in 60 seconds) were assessed. This type of assessment has been validated against more detailed techniques [32], [33], may predict outcome [34], and has been used in the study of CKD patients [13], [35], [36].

### Ethics

Appropriate ethical approval was granted by the South Derbyshire Local Research Ethics Committee and all patients provided written informed consent.

### Statistical methods

Group data are presented as mean ± SD unless otherwise stated. All data were tested for normality. Analysis was performed using SPSS v12.0.1 (SPSS Inc, Chicago, IL). Categorical data was compared using Chi-square test, continuous data using paired or unpaired Students t-test or one-way ANOVA with Tukey's correction as appropriate. Significant alteration in muscle CSA and function (STS-60 and STS-5) was defined as a 5% change.

## Results

### Demographics

88 patients completed 2 year follow up (32 CKD, 40 HD and 16 PD). Their baseline demographics are presented in Table 1. Causes of patient non-completion (46) were primarily death (21), as well as study withdrawal (11) and non-attendance for all 3 annual CT scans (14). Baseline muscle CSA (MCSA) was not significantly different in those who died (10156±2724 vs 10328±2582 mm^2^; p = 0.8), nor did change in muscle mass during the first 12 months predict death during the second year.

**Table 1:** Baseline demographics for cohort.

	CKD (32)	PD (16)	HD (40)	Sig.
Age (years)	62±12	63±11	59±15	ns
Male	17 (53%)	8 (50%)	27 (68%)	ns
Diabetes mellitus	5 (16%)	4 (25%)	10 (25)	ns
Smoking	4 (13%)	1 (6%)	5 (13%)	ns
Dialysis vintage (months)	-	33±22	38±25	ns
BMI (kg/m^2^)	27±6	25±3	27±5	ns
Urea (mmol/l)	23±8	21±5	19±4	0.02, 0.01**
eGFR (MDRD) (ml/min)	16±5	-	-	n/a
Dialysis adequacy (Kt/V)^a^	-	2.44±0.52	1.25±0.35	n/a
Previous CV morbidity^b^	8 (25%)	6 (38%)	7 (18%)	ns
Total cholesterol (mmol/l)	5.2±0.9	4.6±1.0	4.4±1.0	0.002, ns*,***, 0.02**
HDL cholesterol (mmol/l)	1.4±0.3	1.4±0.3	1.3±0.3	ns
LDL cholesterol (mmol/l)	2.8±1.1	2.8±1.1	2.0±0.8	0.008, ns*,***, 0.012**
Triglycerides	1.9±1.2	2.2±0.8	2.4±1.8	ns
Serum phosphate (mmol/l)	1.46±0.3	1.58±0.2	1.73±0.4	0.005, ns*,***, 0.03**
Serum corrected calcium (mmol/l)	2.40±0.1	2.54±0.1	2.47±0.1	0.001, 0.01*, ns**,***
Calcium×Phosphate product (mmol^2^/l^2^)	3.50±0.7	4.01±0.7	4.28±1.0	0.001, ns*,***, 0.001**
PTH (pg/ml)	185±103	362±333	305±318	ns
Albumin (g/l)	34±3	28±3	34±3	<0.001, <0.001*,***, ns**
High-sensitivity C-reactive protein (mg/l)	4.11±5.9	4.02±4.7	8.24±10	ns
Bicarbonate (mmol/l)	23±4	27±3	23±3	<0.001, 0.002*, ns**, 0.001***
Use of Vitamin D	10 (31%)	11 (69%)	22 (55%)	0.026
Lipid lowering therapy	11 (34%)	7 (44%)	13 (33%)	ns
Use of non-Calcium containing binders	1 (3%)	10 (63%)	20 (50%)	<0.001
Use of Calcium containing binders	13 (41%)	6 (38%)	25 (63%)	ns
Use of Calcium channel blockers	13 (41%)	5 (31%)	12 (30%)	ns
Use of ACE inhibitors	15 (47%)	7 (44%)	6 (15%)	0.006
Use of Beta blockers	13 (41%)	2 (13%)	12 (30%)	ns
Use of Epo	7 (22%)	13 (81%)	38 (95%)	<0.001

Results are mean ± SD or number of observations (percentage) for categorical data.

Key/Notes :

ns – not significant.

* – comparison CKD vs PD.

** – comparison CKD vs HD.

*** – comparison PD vs HD.

a – Kt/V is Weekly in PD, per session in HD.

b – defined as any previous description of ischaemic heart disease, heart failure, cerebrovascular disease or peripheral vascular disease.

### Muscle cross-sectional area

There was significant difference in percentage change in MCSA during year 1 dependant on baseline treatment modality (χ^2^ = 6.46; p = 0.039). More CKD patients lost muscle mass (47%) than PD (25%) or HD (35%).

MCSA change during year 1 was not predictive of change during year 2 (Table 2). Whilst, as a group, MCSA decreased over the study (−4.3±8.0%; p<0.001), 7 patients gained MCSA and over 50% (45 of 88) remained static. These observations were not dependent on baseline modality (CKD −5.0±7.1; PD −4.3±8.4; HD −3.8±8.6%).

**Table 2:** Change in MCSA, Year 1 vs. Year 2 for the entire cohort.

		Year 2			
		Lose	No change	Gain	Total
Year 1	Lose	4	13	11	28
	No change	19	28	4	51
	Gain	5	4	0	9
Total		28	45	15	88

χ2 = 14.1; p<0.001.

15 CKD patients started dialysis during year 1 (10 HD, 5 PD). There were few demographic or biochemical differences between dialysis initiators and those remaining without renal replacement therapy (Table 3). Incident dialysis patients overall lost MCSA during the year which they commenced dialysis, but not in the subsequent year (Year 1 −4.4±6.0%, p = 0.01; Year 2 −1.5±10.1%, p = ns)(Tables 4 and 5). Change during year 1 and year 2 was weakly inversely correlated (R = −0.52; p = 0.047). Although there were no differences in year 1 MCSA change by subsequent dialysis modality, recovery of MCSA in year 2 was significantly more common among patients commencing HD (HD 3±7%; PD −10±10%; p<0.01).

**Table 3:** Baseline demographics of Year 1 dialysis initiators (CKD cohort).

	Remained non-RRT (17)	Dialysis initiators (15)	Sig.
Age (years)	63±12	60±12	ns
Male	9 (53%)	8 (53%)	ns
Diabetes mellitus	2 (12%)	3 (20%)	ns
Smoking	2 (12%)	2 (13%)	ns
BMI (kg/m2)	28±6	26±6	ns
Urea (mmol/l)	20±7	28±8	0.004
eGFR (MDRD) (ml/min)	19±4	12±2	<0.001
Previous CV morbidity^b^	3 (18%)	5 (33%)	ns
Total cholesterol (mmol/l)	5.2±0.6	5.2±1.1	ns
HDL cholesterol (mmol/l)	1.3±0.3	1.5±0.4	0.038
LDL cholesterol (mmol/l)	2.8±1.2	2.8±1.1	ns
Triglycerides	2.2±1.3	1.6±1.1	ns
Serum phosphate (mmol/l)	1.31±0.2	1.64±0.2	<0.001
Serum corrected calcium (mmol/l)	2.41±0.1	2.38±0.1	ns
Calcium×Phosphate product (mmol^2^/l^2^)	3.14±0.5	3.90±0.6	0.001
PTH (pg/ml)	135±130	187±143	0.026
Albumin (g/l)	34±3	34±3	ns
High-sensitivity C-reactive protein (mg/l)	3.41±6.3	1.32±3.5	ns
Bicarbonate (mmol/l)	24±4	22±3	ns
Use of Vitamin D	5 (29%)	4 (27%)	ns
Lipid lowering therapy	4 (24%)	7 (47%)	ns
Use of non-Calcium containing binders	1 (6%)	2 (13%)	ns
Use of Calcium containing binders	3 (18%)	8 (53%)	0.027
Use of Calcium channel blockers	8 (47%)	5 (33%)	ns
Use of ACE inhibitors	10 (59%)	5 (33%)	ns
Use of Beta blockers	7 (41%)	6 (40%)	ns
Use of Epo	2 (12%)	5 (33%)	ns

**Table 4:** Change in MCSA, Year 1 vs. Year 2 for CKD patients who remained non-RRT.

		Year 2			
		Lose	No change	Gain	Total
Year 1	Lose	0	4	4	8
	No change	4	2	1	7
	Gain	0	2	0	2
Total		4	8	5	17

χ2 = 3.02; p = 0.082.

**Table 5:** Change in MCSA, Year 1 vs. Year 2 for dialysis initiators.

		Year 2			
		Lose	No change	Gain	Total
Year 1	Lose	1	3	3	7
	No change	4	2	2	8
	Gain	0	0	0	0
Total		5	5	5	15

χ2 = 1.5; p = 0.221.

All demographics, baseline CT imaging, year 1 change in CT imaging and year 1 bloods were examined as univariate predictors of dialysis initiators change in MCSA in year 2. All identified variables with a p-value less than 0.2 (Table 6) were then subjected to a stepwise multivariable linear regression analysis. This identified baseline MCSA, change in MCSA in year 1 and dialysis modality as predictors of change in MCSA in Year 2 (adjusted R^2^ = 0.77; p<0.001) (Table 7).

**Table 6:** Univariate associations in dialysis initiators for change in MCSA during Year 2.

Variable	R	Sig.
Year 1 Modality	0.655	0.008
Diabetes	0.424	0.115
Year 1 Smoker	0.499	0.058
Year 1 Urea	−0.522	0.046
Baseline MCSA	−0.375	0.169
Year 1% change MCSA	−0.520	0.047

**Table 7:** Stepwise LR for dialysis initiators – dependent variable change in MCSA during year 2.

	Unstandardized Coefficients		Standardized Coefficients		
	B	Std. Error	Beta	T	Sig.
(Constant)	−.239	.105		−2.281	.043
Dialysis Modality (HD)	.131	.027	.633	4.878	.000
Year 1 change in MCSA (%)	−.989	.218	−.595	−4.546	.001
Baseline MCSA (mm^2^)	−1.540E-05	.000	−.322	−2.445	.033

There was no significant correlation between change in muscle or fat CSA and other factors including age, gender, inflammatory status, time-averaged bicarbonate, calcium, phosphate or change in serum albumin.

### Muscle function

MCSA correlated with both STS60 and STS5 at all assessments (baseline R = 0.45, p<0.001; R = −0.45, p<0.001; 12 months R = 0.55, p<0.001; R = −0.50, p<0.001; 24 months R = 0.47, p<0.001; R = −0.47, p<0.001). However, MCSA only contributed ∼12% to the observed functional variability at baseline (STS60 Adj R^2^ = 0.11, p = 0.003; STS5 Adj R^2^ = 0.14, p<0.001). In multi-variate modelling MCSA was displaced by age and gender.

There was significant individual variability in the change in both muscle power and endurance during year 1 and 2 (Tables 8 and 9). Change in MCSA correlated poorly with changes in functional ability. STS60 and STS5 changes were dependent on modality. Most variability observed in STS60 was in the CKD (χ^2^ = 10.3, p = 0.001), rather than dialysis groups (PD χ^2^ = 0.7, p = 0.779; HD χ^2^ = 2.4; p = 0.122), and was not solely in those commencing dialysis during the study. STS5 variability was also more marked pre-dialysis, but was more consistent throughout the cohort (CKD χ^2^ = 3.0, p = 0.085; PD χ^2^ = 2.8, p = 0.091; HD χ^2^ = 1.8, p = 0.176).

**Table 8:** Change in STS60 function in year 1 and year 2.

		Year 2			
		−	0	+	Total
Year 1	−	1	1	11	13
	0	1	1	4	6
	+	10	6	9	25
Total		12	8	24	44

(− decrease, 0 no change, + increased).

χ2 = 7.7; p = 0.005.

**Table 9:** Change in STS5 function in year 1 and year 2.

		Year 2			
		−	0	+	Total
Year 1	−	8	1	12	21
	0	2	0	1	3
	+	13	4	2	19
Total		23	5	15	43

(− decrease, 0 no change, + increased).

χ2 = 6.8; p = 0.009.

Although MCSA fell over the study, endurance improved (STS60 +16±9%; p = 0.001), despite no differences in anaemia status.

## Discussion

Whilst we [13] and others have previously documented cross-sectional associations with muscle mass, prospective evaluation has largely been only in intervention based studies. Such control groups that existed in the small number of these studies that were RCTs are limited in number and by the selection bias of the patient demographic agreeing to take part in exercise based studies. This therefore is the first report of the natural progression of both muscle area and function in CKD. This cohort was unselected, not exposed to any kind of exercise regime and followed up for a sufficiently long period to constitute a valid natural history study. Furthermore it is the first study to contain prospective comparative data from well matched patients with non dialysis requiring CKD, HD and PD.

The prevailing view has been that muscle wasting is an inexorable consequence of CKD, and that once commenced, progression is inevitable without intervention. Whilst this cohort did lose muscle mass over the entire study period, the marked variability between years 1 and 2 suggest that even without intervention decline is not continuous. Furthermore, the significant proportion who did not decline over the study affirms that muscle wasting is not inevitable in CKD. During the time frame of this study our patients were not routinely provided exercise advice. Significant muscle wasting is known to occur in acute illness, thus it is possible that the variability seen is in part related to episodic catabolism not assessed in our study – the lack of association with CRP reflecting assessment of baseline inflammatory status rather than cumulative inflammatory load during the preceding year. The role of acidosis is well documented in muscle wasting, yet we did not demonstrate association with serum bicarbonate in this study. We hypothesise that this reflects the low overall levels of systemic acidosis in our cohort.

The increased prevalence of muscle wasting was seen in our pre-dialysis cohort occurs despite all three (CKD, HD, PD) groups being well matched for age, gender, diabetes prevalence, smoking and body mass index (BMI). This observation is somewhat counter-intuitive as CKD 4 patients are usually considered to be less metabolically challenged than CKD 5 patients receiving dialysis. Whilst our CKD 4 cohort were not characterised by a delayed start of RRT (mean eGFR 18 ml/min), they were however significantly more uraemic than the dialysis cohort. This significant loss of muscle CSA in late stage CKD 4 suggests that these patients are having increased disturbance of the catabolic/anabolic balance, presumably as a result of advancing uraemia, and that we are in danger of allowing them to loose ground in these late stages by failing to appreciate this by biochemical changes alone and failing to optimally time dialysis initiation. This raises the possibility that we might be able to utilise this acceleration of muscle wasting as an individualised biomarker of metabolic decompensation, providing a rationale for when the appropriate time to start dialysis might be. This would be supported by our observations in dialysis initiators, in whom the expected acceleration of muscle wasting when clinical deterioration of renal function resulted in the necessary commencement of dialysis was not seen. It is likely that the observed improvement relates to an improvement in the uraemic milieu with the institution of renal replacement therapy, either via direct effects on muscle structure and function, or secondary to improvement in subjects' activity promoting the rebuilding of muscle mass.

The differential effects of modality in the dialysis initiators are challenging to explain. Indeed, some caution is required in interpretation as this group is relatively small and not randomised. However, dialysis modality was a free choice for participants and there were no significant differences in age, gender or other demographics, baseline renal function or inflammatory status.

Both insulin resistance [19], [20] and plasma insulin concentration [19] are associated with muscle breakdown in non-diabetics. Diabetic dialysis patients demonstrate increased muscle breakdown c.f. non-diabetics [9], [10]. Glucose-based PD therapy causes hyperglycaemia and hyperinsulinaemia even in non-diabetics [37]. It is thus possible that these observed differences relate to the deleterious effects of intraperitoneal glucose.

Our original findings of lower MCSA in dialysis patients [13] are consistent with this study demonstrating pronounced muscle loss pre-dialysis which is improved with dialysis. We hypothesise that significant muscle loss is occurring in the final period before renal replacement therapy is commenced, and whilst this is improved with commencement of dialysis, patients do not return to pre-existing ‘CKD’ levels. We did not see a relationship between dialysis vintage and MCSA in our dialysis population in this study, but do acknowledge that this may, in part, relate to the inherent selection bias of the requirement of repeated assessments in this study.

Whilst both endurance and power correlate with MCSA, this represents only a relatively small contribution. There are thus clearly other factors than muscle CSA affecting function, an observation which is consistent with Kemp et al's [2] findings of lower MCSA, but normal contractile efficiency, in HD. Cheema et al [27] showed no statistically significant change in muscle CSA after exercise training, but a highly significant increase in muscle function (both power and endurance), supporting this observation that CSA is only a component of total functional ability.

However, detailed further analysis of our cohort did not demonstrate other consistent predictors of changes in function. It is well documented that muscle structure affects function, and thus it is a limitation of our study that muscle biopsies were not performed. However, this study was designed to investigate the relationship between muscle function and *clinically* relevant, readily obtainable markers in common use.

Variability in MCSA could represent a lack of reproducibility between scans. In part, this was the reason for the selection of 5% as a cutoff for significant change, rather than a lower one. It would not have been ethically appropriate to repeat CT assessment, and it must be remembered that CT is a test in clinical use, with reproducibility sufficient for clinical surveillance. Furthermore, scan location was anatomically defined and care was taken during analysis to ensure that the same area was being assessed on follow-up scans. Cross-sectional CT has also been used in other studies of muscle CSA [27], [38]. However previous studies have relied on single mid-thigh measurements, whilst we have taken the average of 3.

Lack of relationship with age, gender, inflammatory status or albumin is notable as there were significant differences with these variables in our previous cross-sectional baseline report of this cohort. It is thus likely that whilst they are associated with current total muscle mass either changes in these factors do not determine changes in muscle mass or they do so within a time frame beyond the scope of this study. Cheema et al [27] found a decrease in log CRP with exercise, but we did not demonstrate any relationship between change in MCSA and either cross-sectional or change in inflammatory status. This was however a population defined by a relatively low prevalence of systemic inflammation. This lack of significant association between changes in these predictors and changes in MCSA underlines that the pathophysiology involved is poorly understood, and may suggest that the effect of the non-modifiable factors (age, gender) coupled with the variability in other factors (eg. exercise, dialysis modality, vascular change), overwhelm these changes. Alternatively, the magnitude of effect may simply leave our study underpowered to detect this.

## Limitations

We acknowledge that we do not present data regarding protein intake, insulin resistance or activity data. However, patients in our unit were provided with routine dietetic advice as part of their clinical care during this study, we did not recommend protein restriction. Participants undertook normal activity levels during the study.

We also acknowledge the number of participants who did not complete the study. This reflects in part the high attrition rate of CKD patients (accepting that generally a ‘fitter’ subgroup will volunteer for clinical studies) combined with volunteers maintaining the significant time commitment often needed to participate in clinical research studies.

## Conclusion

We have demonstrated variability in the natural course of muscle wasting in CKD, most marked pre-dialysis with improvement on dialysis. Change in muscle mass is not predicted by factors associated cross-sectionally, and poorly predicts function. Monitoring for accelerated muscle loss might represent an attractive patient specific ‘bioassay’ for the need to commence renal replacement therapy. This approach appears to warrant further investigation.
